# Clinical Potential of Mesenchymal Stem Cell-Derived Exosomes in Bone Regeneration

**DOI:** 10.3390/jcm12134385

**Published:** 2023-06-29

**Authors:** Bárbara Torrecillas-Baena, Victoria Pulido-Escribano, Gabriel Dorado, María Ángeles Gálvez-Moreno, Marta Camacho-Cardenosa, Antonio Casado-Díaz

**Affiliations:** 1 Unidad de Gestión Clínica de Endocrinología y Nutrición-GC17, Instituto Maimónides de Investigación Biomédica de Córdoba (IMIBIC), Hospital Universitario Reina Sofía, 14004 Córdoba, Spain; b42tobab@uco.es (B.T.-B.); victoriapulido7@gmail.com (V.P.-E.); mariaa.galvez.sspa@juntadeandalucia.es (M.Á.G.-M.); 2 Department Bioquímica y Biología Molecular, Campus Rabanales C6-1-E17, Campus de Excelencia Internacional Agroalimentario (ceiA3), Universidad de Córdoba, 14071 Córdoba, Spain; bb1dopeg@uco.es; 3 CIBER de Fragilidad y Envejecimiento Saludable (CIBERFES), 14004 Córdoba, Spain

**Keywords:** regenerative medicine, bone, mesenchymal stem cells, extracellular vesicles, exosomes, biomaterials, cell-free therapy

## Abstract

Bone metabolism is regulated by osteoblasts, osteoclasts, osteocytes, and stem cells. Pathologies such as osteoporosis, osteoarthritis, osteonecrosis, and traumatic fractures require effective treatments that favor bone formation and regeneration. Among these, cell therapy based on mesenchymal stem cells (MSC) has been proposed. MSC are osteoprogenitors, but their regenerative activity depends in part on their paracrine properties. These are mainly mediated by extracellular vesicle (EV) secretion. EV modulates regenerative processes such as inflammation, angiogenesis, cell proliferation, migration, and differentiation. Thus, MSC-EV are currently an important tool for the development of cell-free therapies in regenerative medicine. This review describes the current knowledge of the effects of MSC-EV in the different phases of bone regeneration. MSC-EV has been used by intravenous injection, directly or in combination with different types of biomaterials, in preclinical models of bone diseases. They have shown great clinical potential in regenerative medicine applied to bone. These findings should be confirmed through standardization of protocols, a better understanding of the mechanisms of action, and appropriate clinical trials. All that will allow the translation of such cell-free therapy to human clinic applications.

## 1. Introduction

Bone is a complex organ providing mechanical support, locomotion, soft-organ protection, and endocrine functions. It is composed of organic components, such as type I collagen, glycosaminoglycans, and glycoproteins, and inorganic components, predominantly phosphate and calcium ions. Other inorganic components like sodium, bicarbonate, potassium, citrate, magnesium, carbonate, fluorite, zinc, barium, and strontium can also be found [1,2]. Another important bone function is to harbor the bone marrow. It is situated in axial and long bones, and even in certain spaces within spongy bones. The bone marrow provides a niche where numerous types of stem cells reside. Among them, the mesenchymal stem cell (MSC) and hematopoietic stem cell (HSC) populations stand out. The former are progenitors of osteoblasts, chondrocytes, and adipocytes, among other cell types; the latter give rise to cells of lymphoid and myeloid lineages [1,3].

There are three main types of cells in bone: osteoblasts, osteocytes, and osteoclasts. The first ones are cuboidal cells, situated along the bone surface, and known for their bone-forming functions. During bone formation, a subpopulation of osteoblasts undergoes morphological changes, becomes surrounded by mineralized extracellular matrix and differentiates into osteocytes. They are incorporated into the bone matrix, interconnecting via canaliculi and cytoplasmic protrusions. That allows the transference of molecules, nutrients, and hormones. Osteocytes are capable of detecting mechanical pressures and loads, allowing the adaptation of bone to mechanical forces [1]. On the other hand, osteoclasts come from the merger of several monocytes and are responsible for bone resorption [2].

Bone is highly vascularized and innervated. Blood vessels are essential for bone homeostasis. They deliver oxygen, nutrients, and hormones. Besides, they can transport immune and precursor cells to and from bone, including bone marrow [4]. Furthermore, a lot of nerve fibers are situated along the bone. They can transmit signals to muscle and tendon tissues, which can influence bone stretching or compression. Moreover, peptidergic nerves can release some neurogenic factors such as neuropeptide Y (NPY), substance P (SP), and calcitonin gene related peptide (CGRP) that bind to skeletal cell receptors, promoting bone regeneration and maintaining bone homeostasis [5].

Bone is a particularly dynamic organ, in which old tissue is constantly replaced by new tissue. This process is known as remodeling, involving bone resorption by osteoclasts and bone formation by osteoblasts [6]. It is a very important process to replace and repair deteriorated bone tissue, maintain calcium homeostasis, and further maintain the appropriate mechanical characteristics of the skeleton. There must be a balance between bone formation and resorption, which is regulated by some factors like cytokines, chemokines, hormones, and biomechanical stimulation [2]. Such equilibrium may be lost with aging or certain pathological conditions, favoring bone resorption over bone formation. This may lead to abnormal bone remodeling and the development of bone diseases such as osteoporosis, osteoarthritis, and fractures. The latter may be the result of bone fragility, due to loss of bone mass or trauma [6]. It has been estimated that a total of 15 million fractures occur per year worldwide, representing a significant socio-economic cost. Thus, bone repair and regeneration has attracted extensive interest [7].

In recent years, regenerative medicine has aroused great interest for the treatment of bone defects, whether associated with aging or not. Furthermore, research in bone regeneration has grown in the last few years, thanks to the discovery of new nanomaterials and improvements in technology in the field of biomedicine [7]. Several studies have focused on the use of cell therapy, for the enhancement of bone tissue regeneration. Cell therapy is mainly based on the use of MSCs, due to their differentiation and regenerative capacity [8]. However, implementing this type of therapy presents challenges and limitations. That is due to the difficulty of maintaining optimal cell potency and viability during cell expansion, growth, and delivery to the patient. In addition, interestingly, recent studies have revealed that MSC potential in tissue regeneration is due to their paracrine effects [9]. Therefore, the use of secretomes from these cells is becoming a good alternative to cell therapy in regenerative medicine [10]. This review is a summary of the current information on the topic. It shows that extracellular vesicle (EV) fractions, derived from MSC secretomes, have a high therapeutic potential in bone regeneration.

## 2. Physiological Processes in Bone Regeneration

Mineralized bone structures contain cells, vessels, and crystals of calcium phosphate (mainly hydroxyapatite) [11]. Bone remodeling is a dynamic process in which old bone is replaced by new tissue. That is accomplished through the interaction of different cell phenotypes involved in the maintenance of skeletal integrity [12]. Thus, osteoblasts are responsible for production of bone matrix. They originate from differentiating multipotent-mesenchymal stem cells [13]. When osteoblasts mature, they become entrapped in new bone matrix, differentiating into osteocytes or remaining on the bone surface, becoming flat-lining cells [11]. On the other hand, osteoclasts derive from mononuclear hematopoietic cells, specifically from the monocyte-dendritic-macrophagic lineage [14]. They are responsible for bone resorption by digesting the mineralized cellular matrix [15]. In this sense, bone cells are organized in a complex called basic multicellular units (BMU), in which bone reabsorption and formation are coupled [11]. A balance between these mechanisms is essential to repair defects due to mechanical load and strain, preventing aging effects [12].

Bone-regeneration cycles begin with the activation of osteocytes and preosteoblasts in the bone marrow, which secrete receptor activator of NF-kappa B ligand (RANKL) on the bone surface. Such a protein, a member of tumor necrosis factor (TNF), interacts with receptors present on osteoclast precursors, called receptor activator of NF-kappa B (RANK). That promotes activation, differentiation, and fusion of hematopoietic cells of the osteoclast linage and, therefore, bone resorption [16]. Osteoblasts, as well as other cells of the bone marrow, produce osteoprotegerin (OPG), inhibiting the final differentiation and activation of osteoclasts [17]. Then, the remodeling cycle continues with a reversal phase. Thus, mononuclear cells appear on the bone surface, preparing it for the formation, differentiation, and migration of new osteoblasts [11]. In the last phase of bone regeneration, its surface is covered with flattened lining cells. A rest period (or quiescence phase) occurs before a new remodeling cycle [12].

During remodeling, vascularization of bone tissue is essential, providing a source of adult mesenchymal stem and other osteoprogenitor cells as well as necessary nutrients for mineralization and calcium mobilization [18]. In this sense, vascular endothelial growth factor (VEGF) has been suggested as the osteogenic-angiogenic coupling factor [19], regulating the expansion and survival of mesenchymal progenitors. VEGF signaling is also coupled to morphogenetic, metabolic, inflammatory, and mechanical cues, controlling mineral metabolism [18]. Previous studies have demonstrated that different factors represent rate-limiting components of osteogenic-angiogenic coupling and bone formation. They include osteoblast hypoxia-inducible factor alpha (HIFA) subunits, transcriptional regulators of VEGF, and erythropoietin expression [20]. VEGF can expand VEGF receptor 2 (VEGFR2)-expressing mesoangioblasts during angiogenesis. Thus, this process may increase osteoblasts, promoting bone formation in the osteogenic marrow environment [21].

Injury-induced hematoma and inflammation begin in the first steps of fracture healing, in order to promote the accumulation of mesenchymal stem cells. They will differentiate along the chondrocyte and osteoblast lineages [22]. Then, healing mechanisms continue with the production of chondrocytes, which form cartilaginous scaffolds. They expand to bridge fractured ends and then mineralize to form rigid-hard calluses. Angiogenesis plays an important role in this phase. Indeed, its inhibition may impair callus formation, compromising bone fracture healing [23,24].

Until normal bone geometry is re-established, hard calluses are remodeled by the coordinated actions of osteoclasts and osteoblasts [25]. Estrogen loss increases osteoclasts and decreases osteoblasts. That may lead to an unbalanced activity of BMU in favor of bone resorption, based on the unitary model for the pathophysiology of osteoporosis [26]. In addition, previous studies have evidenced the role of senescent cells, which increase during aging. Among the most important features of MSC senescence, functional alterations caused by metabolic, genetic, epigenetic, transcriptional, and translational changes have been described [27]. These cells also acquire a senescence-associated secretory phenotype (SASP) that affects neighboring cell behaviors via paracrine mechanisms [28]. Given the complex nature of SASP, several biological processes such as cell proliferation, angiogenesis, inflammation, wound healing, and other types of tissue repair are affected by this paracrine response [29]. Furthermore, vascularization is impaired in aging donor derived MSC due to decreased secretion of proangiogenic factors (including VEGF), as well as increased secretion of anti-angiogenic factors and factors involved in ECM remodeling [30].

Furthermore, previous studies have demonstrated the critical role of EV, and in particular their non-coding RNA composition, in the regulation of target cells by MSC during aging [31]. Certain miRNAs within exosomes secreted by young MSC can suppress the cellular aging of hematopoietic stem cells. On the other hand, vesicles from senescent MSC may significantly aggravate this process [32]. Therefore, during aging, the accumulation of senescent MSC may result in an impairment of stem cell functions, altering tissue maintenance and regeneration [33]. That may generate tissue dysfunction and, therefore the development of several age-related diseases, such as osteoporosis [34]. Moreover, common features of senescent cells include the induction of local inflammation and the attraction of immune cells. That is generated by proinflammatory cytokines and chemokines, which are components of the SASP and are largely conserved in all cell types [29,35]. Indeed, these senescent cells can be characterized by the excessive production of proinflammatory cytokines. Therefore, “inflammaging” phenomena in elderly people have been linked to bone loss and fracture risk [16]. Thus, therapies involving modulation of inflammation as well as promotion of the expression of genes encoding anti-inflammatory factors may have a positive impact on bone regeneration. For the development of cell therapy strategies, the origin of MSC from both aged donors and senescent-culture media must also be taken into account to avoid the above-mentioned effects of senescent cells.

## 3. Mesenchymal Stem Cells in Bone Regeneration

Bone regeneration is a major challenge for patients with impaired tissue healing capacity due to aging and/or various diseases. So, bone tissue repair is of great relevance, both clinically and economically. Mesenchymal stem cells, or MSC-based tissue engineering strategies, have been used to improve bone regeneration for years. Recently, the focus has broadened to cell-free therapies, exploiting the paracrine effects of MSC [10].

As previously mentioned, bone remodeling is a key process in bone formation and homeostasis, in which osteoblasts, osteoclasts, and osteocytes are involved. In addition to these cell types, bone remodeling is also modulated by MSCs from the bone marrow. Indeed, MSC are precursors of osteoblasts and regulate osteoclasts. This gives MSC great relevance for the regeneration of bone tissue [36]. MSC are multipotent cells with fibroblastic morphology, first described in 1970 [37]. The minimum criteria for defining MSC, established by the Mesenchymal and Tissue Stem Cell Committee, of the International Society for Cellular Therapy (ISCT), are: (i) plastic-adherent; (ii) express CD73, CD90, and CD105; (iii) lack expression of CD11b, CD14, CD19, CD34, CD45, or CD79a and HLA-DR surface molecules; and (iv) be able to differentiate into osteoblasts, adipocytes, and chondroblasts in vitro [38].

MSC are considered a powerful tool in regenerative medicine, due to their high capacity for proliferation, differentiation, and modulation of physiological processes. They include hematopoiesis, inflammation, and angiogenesis, as well as their low immunogenicity. The sources of MSC are varied, with the main one being bone marrow. Some clinical studies have shown that MSC from different origins may have the capacity to repair injured tissues [39].

When bone damage or fracture occurs, MSC are capable of migrating towards injury sites to promote tissue regeneration. Various studies have demonstrated the possible contributions of MSC to skeletal homeostasis. Indeed, they represent a potential therapeutic resource for the treatment of bone diseases [36]. It has recently been shown that the regenerative capacity of MSCs is due to their paracrine effect. The use of media enriched in cytokines and factors secreted by MSC is an alternative to therapies involving cells. Indeed, the advantages of using cell-free secretomes over cell- and tissue-based therapies are significant. For instance, the absence of allogeneic cells, as well as low metabolic activity, improves patient safety and quality control [40].

MSC secretomes are composed of soluble factors and EV. The latter are nano-sized particles secreted by cells. They may exert different pleiotropic effects on various cellular processes, can be released by the majority of cell types, and can be found in a variety of fluids [41]. Different studies in animal models have shown the therapeutic power of MSC secretomes in bone regeneration [41,42,43,44].

Additionally, bioactive scaffolds are frequently used as materials to promote bone regeneration. These scaffolds provide temporary mechanical support for bone and are biodegradable. Moreover, scaffolds are three-dimensional and highly porous structures. That favors cell proliferation, providing space for new tissue growth and vascularization [45,46]. Furthermore, encapsulation of MSC or EV in scaffolds may enhance osteoinduction in clinical applications. Thus, a biomaterial was created combining 3D-printed poly(ε-caprolactone)/nano-hydroxyapatite scaffolds with hydroxypropyl chitin-based hydrogels, in which MSC were encapsulated. The resulting biomaterial significantly increased bone formation in a rat calvarial defect model with respect to both the control and the biomaterial without MSC. Therefore, this study showed that MSC enhanced osteoinductive processes. In addition, the authors also demonstrated that encapsulated MSC promoted bone formation. That was accomplished through their differentiation into osteoblasts and the secretion of factors. The latter created microenvironments suitable for promoting osteogenesis and angiogenesis. That highlights the importance of the paracrine activities of MSC in tissue regeneration [47]. As a consequence, instead of encapsulating MSC in this type of material, there is a trend to use cell-free systems, in which MSC-EV are incorporated, for steady, sustained, and localized release into damaged tissues. That favors regeneration, as described in the following sections of this review.

## 4. Extracellular Vesicles: Properties and Therapeutic Potential

Extracellular vesicles are heterogeneous particles enclosed by a lipid membrane. They can carry a wide variety of biologically active molecules [48]. EV are released by the majority of cells, including MSC [49], embryonic stem [50], dendritic [51], T and B [52,53], epithelial [54], endothelial [55], mast [56], oligodendrocytes [57], neuronal [58], Schwann [59] and cancerous ones [60]; and even cell fragments like platelets [61]. Furthermore, EV can be isolated from some fluids like urine, bile, blood, breast milk, saliva, semen, other fluids (amniotic, synovial, nasal, bronchoalveolar, and cerebrospinal), and malignant ascites [8,41,62].

There are several types of EVs that are classified according to their size, shape, content, release mode, and function. One of the most commonly used classifications divides them into microvesicles, exosomes, and apoptotic bodies. Microvesicles sizes vary from 20 to 1000 nm and are formed by the evagination of plasma membranes. On the other hand, exosomes range between 40 and 100 nm and have an endosomal origin. They are released by exocytosis of multivesicular bodies through plasma membranes. Finally, apoptotic bodies are larger in size, ranging from 1000 to 5000 nm, and are released during early apoptosis stages [62,63].

There are different classifications and markers used to characterize EV. The International Society for Extracellular Vesicles stated that EV is the preferred generic term [64]. The most common techniques used for their isolation are filtration, size exclusion, density centrifugation, ultracentrifugation, affinity and immunity separation. In fact, a combination of isolation and purification methods is necessary to obtain quality EV. In the same way, EV characterization can be carried out using techniques like immunoblotting, flow cytometry, transmission electron microscopy (TEM), scanning electron microscopy (SEM), atomic force microscopy (AFM), nanoparticle tracking analysis (NTA), dynamic light scattering (DLS), resistive pulse sensing, enzyme-linked immunosorbent assay (ELISA), and fluorescence-activated cell sorting (FACS), among others [65,66].

The use of EV in regenerative medicine is considered a cell-free therapy. This has several advantages in comparison with cell therapies. For example: (i) easy isolation and storage without loss of cargo potency; (ii) the presence of lipid bilayers gives them stability to avoid degradation of their contents; (iii) can be applied in large doses, infiltrating target organs; (iv) can be intravenously administered, being able to reach the smallest capillaries; (v) ability to cross the blood-brain barrier; (vi) preclude potential tumorigenicity, cellular dedifferentiation, embolism, and immune rejection; (vii) safety, dosage, and potency can be evaluated, like a conventional pharmaceutical product; and viii) cargos can be modified for different and optimized therapeutic applications [63,67,68,69].

EV cargos are composed of different molecules, including peptides, proteins, lipids, nucleic acids [microRNA (miRNA), short interfering RNA or silencing RNA (siRNA), long non-coding RNA (lncRNA), mRNA, DNA, etc.], and carbohydrates. They may exert paracrine effects on receptor cells. EV cargos can trigger functional responses and promote phenotypic and functional changes that can affect recipient cells under physiological or pathological conditions [42,65]. Nevertheless, EV can activate other cell surface receptors without delivering their cargo [64]. Such cargoes can be different depending on cell type, microenvironments, and physiological conditions. For this reason, preconditioning factors such as hypoxia, physical or chemical changes, and pharmacological agents may impact EV production and contents [70].

In the last few years, EVs have acquired great relevance due to their clinical potential. Numerous preclinical studies have been or are currently being carried out, studying pathologies like cardiovascular, neuronal, lung, renal, and liver injuries, in addition to cancer and wound healing [71,72]. Due to their properties, EVs have been applied as diagnostic and therapeutic tools, in clinical fields, especially in relation to cancer. At the time of writing, more than 90 clinical trials were registered in the US-NIH clinical trial database https://clinicaltrials.gov searching, accessed on 28 October 2022, for the term exosomes.

In fact, a lot of studies have been published on this topic. In connection with cancer, for instance, a urine exosome gene expression assay was tested using a noninvasive technique that could discriminate between high grades of prostate cancer and benign disease [73]. Additionally, it has been demonstrated in urine that thyroglobulin in EV could be an important pro-inflammatory predictor and biomarker of thyroid cancer recurrence for certain patients [74]. Moreover, exosomal piwi-interacting RNA (piRNA), like piR-10506469, was analyzed as a potential biomarker for the diagnosis of cholangiocarcinoma and gallbladder carcinoma [75].

On the other hand, neural system-related clinical trials have been conducted using EV. For example, EV could be a relevant method for monitoring diseases such as glioblastoma multiforme, brain metastasis, and meningioma [76]. Additionally, levels of plasma EV Aβ1-42 and tau, in combination with the age and gender of the patients, were found to be highly accurate markers of cognitive dysfunction in Parkinson disease [77].

From a therapeutic point of view, other studies have focused on coronavirus disease 2019 (COVID-19). Thus, it has been demonstrated that inhalation of MSC-derived EV for five days improved chest computed tomography imaging [78]. Additionally, exosomes have been used for the attenuation of chronic airway inflammation [79]. As mentioned above, EVs have considerable clinical potential for the diagnosis or treatment of various pathologies. Among them are those related to bone regeneration, which are the main focus of this review.

## 5. Clinical Potential of Mesenchymal Stem Cell-Derived Extracellular Vesicles in Bone Regeneration

**Figure 1:**
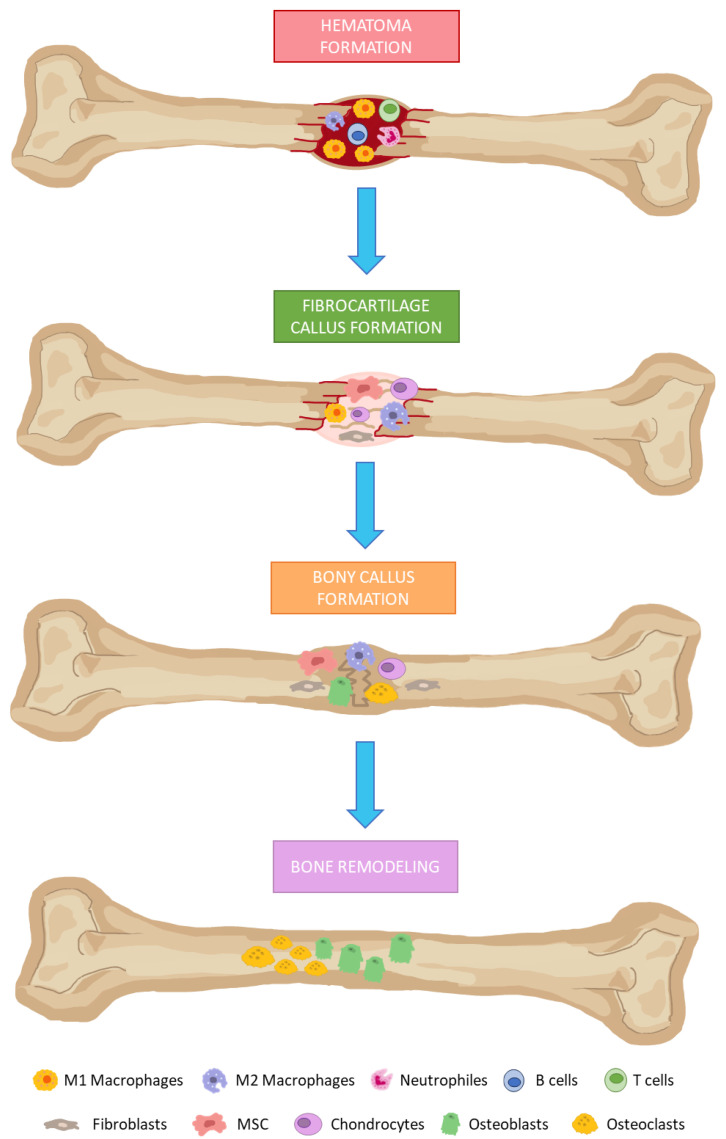
Bone regeneration phases. (1) Hematoma formation: infiltration of neutrophils, macrophages, and lymphocytes. (2) Fibrocartilage callus formation: recruitment of fibroblasts and mesenchymal stem cells. The last one differentiates into chondrocytes at the fracture site. (3) Bony callus formation: differentiation of chondroblasts, chondroclasts, osteoblasts, and osteoclasts is stimulated. Then, cartilaginous calluses are resorbed and begin to calcify. (4) Bone remodeling: osteoblasts and osteoclasts will give rise to successive cycles of remodeling to produce mature bone tissues.

In the presence of bone damage, a series of physiological processes are activated, directed towards bone regeneration. These events occur through a series of consecutive and overlapping phases, including the formation of: (i) hematoma; (ii) fibrocartilaginous callus; and (iii) bony callus; and (iv) bone remodeling (Figure 1) [80]. Hematomas occur immediately after bone fractures as a consequence of ruptured blood vessels. Damaged tissues secrete pro-inflammatory cytokines. That favors the infiltration of neutrophils, macrophages, and lymphocytes. They remove damaged and necrotic tissue in addition to inducing tissue regeneration through the secretion of different growth factors.

Fibrocartilaginous calluses are formed after induction of angiogenesis in the previous phase, and recruitment of mesenchymal stem cells. They will mainly differentiate into chondrocytes at the fracture site, producing a matrix rich in collagen, thus giving rise to fibrocartilaginous tissue. Bony callus formations consist in endochondral ossification of cartilaginous calluses. For this purpose, *RANKL* expression is induced. That way, differentiation of chondroblasts, chondroclasts, osteoblasts and osteoclasts is stimulated. Then, cartilaginous calluses are resorbed and begins to calcify, while angiogenesis and the influx of more MSC progresses. As a result, at the end of this phase, calcified calluses of immature bones are formed. In the bone remodeling phase, osteoblasts and osteoclasts will give rise to successive cycles of remodeling, producing mature bone tissues [80,81].

For the correct development of these phases, there must be adequate coordination among the different cell types participating in each of them. This requires cellular interconnection involving paracrine processes that functionally activate or inhibit cells according to their role in each of the phases of bone regeneration. Part of these cellular communication processes are mediated by the secretion of extracellular vesicles [82]. Due to the variety of bioactive molecules, they can transport, EV acts through all phases of bone regeneration. As noted above, MSCs play a pivotal role in bone formation and regeneration [36].

**Figure 2:**
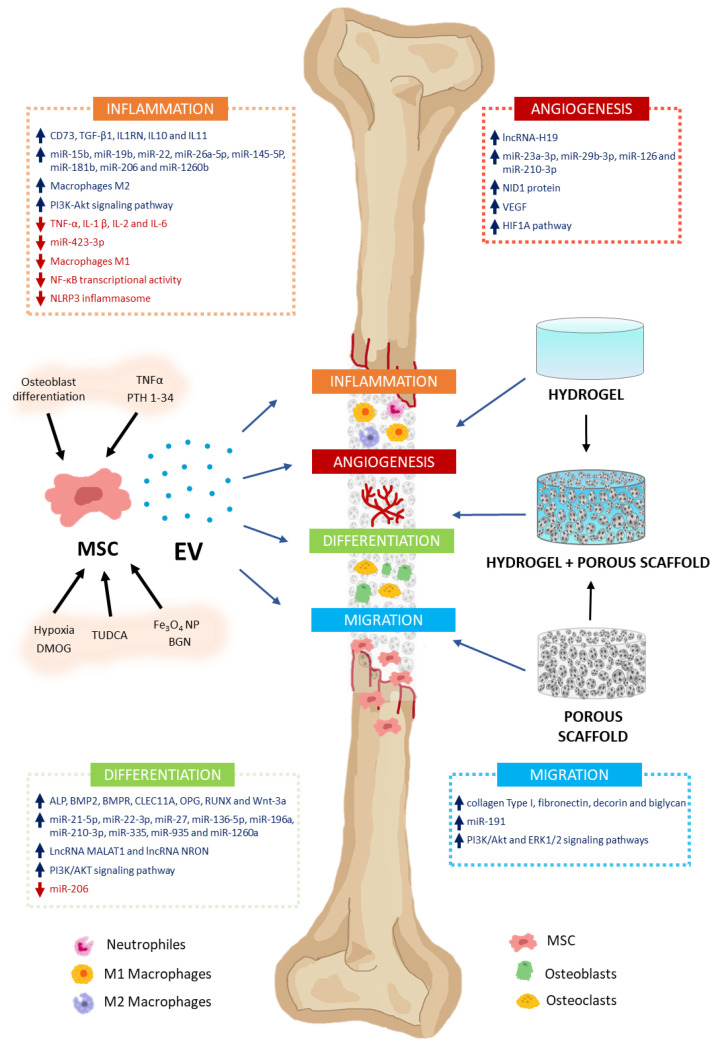
Effects of MSC-EV on bone regeneration. MSC-EV have the capacity to modulate different processes related to bone formation and regeneration. They include inflammation, angiogenesis, differentiation, and cell migration. This is due to their cargos, which have high (**↑**) or low (**↓**) levels of different molecules with biological activity (growth factors, cytokines, miRNA, lncRNA, etc.). They intervene in the induction or inhibition of different signaling pathways related to these processes. To stimulate secretion of EV with high regenerative capacity in MSC cultures, cells can be preconditioned with different molecules [TNFα, PTH 1-34, tauroursodeoxycholic acid (TUDCA), and dimethyloxalylglycine (DMOG)], nanoparticles (Fe_3_O_4_ NP), bioactive glass nanoparticles (BGN), or culture conditions (osteoblast differentiation and hypoxia). MSC-EV can be applied for the treatment of bone defects, incorporating them into biomaterials that serve as vehicles and delivery systems for them. Thus, cell migration, proliferation, and differentiation are facilitated in the scaffolds, enabling bone regeneration. Hydrogels, porous scaffolds, and those resulting from the combination of both are interesting biomaterials with great potential for treating bone damage.

In addition to MSC involvement as osteoblast and chondrocyte precursors, they may also intervene in modulating various processes through the secretion of paracrine factors, among which EV stands out. These processes include inflammation, angiogenesis, cell proliferation, and migration, as well as osteoblast and osteoclast differentiation. That makes MSC-EV a very interesting potential tool in regenerative medicine for bone therapy applications [10] (Figure 2). Thus, in this section, we will describe how MSC-derived EV modulates different processes related to bone regeneration and metabolism.

### 5.1. Inflammation

Upon bone damage, the inflammatory phase is immediately activated, with an increase in inflammatory cytokine levels that peak at 24 h, and gradually decline over the following seven days, according to several studies [83]. If the inflammatory phase is further prolonged, bone regeneration may be delayed [84]. Therefore, numerous studies have paid much attention to the immunomodulatory effect of MSC-EV, promoting bone regeneration. In this regard, it has been observed that polycaprolactone (PCL) scaffolds containing S-nitrosoglutathione and human bone marrow-derived mesenchymal stem cells (hBMSC)-derived EV favor the regenerative capacity of bone pathologies. That is accomplished by favoring the induction of osteoblast differentiation as well as repressing inflammatory genes [85].

Because the contents of EV depend on the physiological conditions of the cells secreting them, MSC culture conditions influence the regenerative capacity of EV. Thus, for example, EV derived from MSC induced to differentiate into osteoblasts for zero, three, and seven days, as opposed to those differentiated for 14 days, repressed the expression of proinflammatory genes in macrophage cultures, in addition to the expression of markers of the M1 inflammatory phenotype. This shows a higher regenerative capacity in MSC- or preosteoblast-derived EV [86].

MSC preconditioning or priming with different compounds is one of the strategies used to enhance the osteogenic properties of MSC-EV (Table 1). Among the compounds used for this purpose is tauroursodeoxycholic acid (TUDCA), which is an amphiphilic molecule implied in transporting lipids, to maintain cholesterol homeostasis. MSC treated with TUDCA had reduced cholesterol levels and increased EV production [87]. In addition, this treatment increased in EV the concentrations of anti-inflammatory cytokines such as interleukin 1-receptor antagonist (IL1RN) and interleukins (IL6, IL10, and IL11), as well as the presence of miR-145-5P anti-inflammatory miRNA, while decreasing the expression levels of miR-423-3p inflammatory miRNA, with respect to MSC-EV from untreated cultures. Besides, MSC-EV derived from TUDCA-treated cultures induced bone repair, in an in vivo model of long bone defects in rats. In part, this was due to an increase in IL10, transforming growth factor beta 1 (TGF-β1) and IL1RN anti-inflammatory cytokines in treated animals [88].

Preconditioning MSC with inflammatory cytokines has also been used as a strategy to enhance immunomodulatory responses of MSC. Thus, EV derived from human MSC cultures treated with 20 ng of tumor necrosis factor alpha (TNFα)/mL, for 72 h, had an increased ability to polarize primary mouse macrophages from the M1 (pro-inflammatory) to the M2 (regenerative) phenotype. Interestingly, the cargos of these EV were enriched in miRNA with anti-inflammatory activity, such as miR-15b, miR-19b, and miR-22, with respect to those of cultures not preconditioned with TNFα. Because of this, their regenerative capacity was also greater, as shown by the fact that their application in a rat calvarial defect model, in addition to reducing inflammation, produced greater bone formation [89].

TNFα has also been used for the preconditioning of gingival tissue derived MSC (GMSC) to enhance their ability to treat periodontitis. This pathology is characterized by inflammation of the periodontium, and subsequent destruction of the alveolar bone supporting teeth. It is one of the main causes of tooth loss in adults [90]. Interestingly, GMSC-derived EV preconditioned with TNFα were enriched in CD73 and miR-1260b. The former reduced inflammation through the induction of M2 macrophage polarization, while the latter inhibited Wnt5a expression (Wnt is a portmanteau of Wg and int and stands for “wingless-related integration site”) and consequently osteoclastogenesis. That was accomplished through downregulation of the RANKL pathway. Therefore, these EVs have a high potential for the treatment of periodontitis [101].

One of the most important inflammatory pathologies related to bone metabolism and joints is osteoarthritis. It is characterized by subchondral bone sclerosis and synovial inflammation. The latter results in synovial hyperplasia, fibrosis, thickening of the synovial capsule, and activated synoviocytes. In addition, it may produce lymphocytic infiltrates (B- and T-cells, as well as plasma cells), cartilage degradation, ligament calcification, and osteophyte formation [102]. The prevalence of this disease in people over 60 years of age is 10% in men and 18% in women [103].

Due to the anti-inflammatory capacity of MSC-EV, their use as a cell-free therapy for the treatment of osteoarthritis has been suggested [104]. In this context, numerous studies have evaluated the effect of MSC-EV on different types of in vitro and in vivo models of osteoarthritis. Thus, treatment with BMSC-EV of equine chondrocytes exposed to inflammatory cytokines, interleukin (IL)-1β and TNF-α, down regulated the gene encoding metalloproteinase 13, which is associated with chondrocyte degeneration in inflammatory situations [105]. Interestingly, intra-articular injection of 2.5 µg of EV derived from BMSC or adipose tissue-derived mesenchymal derived stem cells (AdMSC) improved cartilage regeneration at the histological level. That was observed in a mouse model of osteoarthritis induced by ciprofloxacin. Expression of genes related to chondrocyte markers, such as type II collagen, was upregulated, mainly when the EV were derived from BMSC [103]. In addition to BMSC, human umbilical cord mesenchymal stem cells (hUCMSC) have also been used as a source of EV, in order to treat osteoarthritis. UCMSC have the advantage that they can be easily isolated and do not entail ethical issues because they are derived from tissues considered waste.

On the other hand, UCMSC-derived EV activated macrophage polarization into the M2 phenotype, inducing expression of IL-10 (an anti-inflammatory cytokine) while repressing expression of genes encoding proinflammatory factors such as TNF-α, IL-1, and IL-6. Furthermore, application of these EVs in the articular lumen confirmed their anti-inflammatory effects in a rat model of osteoarthritis. Thus, they increased M2 macrophages and decreased inflammatory cell infiltration [106]. Genomic and proteomic analyses of EV-UCMSC showed that they are enriched in miRNA such as miRlet-7a-5p, miR-100-5p, miR-122-5p, miR-148a-3p, and miR-486-5p, as well as proteins like A2M, ACTB, ALB, HBA1, ANXA2, ANXA6, C3, FN1, HBE1, and MFGE8. Part of these molecules are involved in the regulation of the phosphatidylinositol 3-kinase (PI3K) protein kinase B (PKB; also known as Akt) signaling pathway, which is involved in the activation of M2 macrophages. This explains, in part, the chondroprotective mechanism of EV-HCMSC [106,107]. Interestingly, MSC-EV as a treatment for osteoarthritis also positively affected bone formation. Thus, in a mouse model of temporomandibular joint osteoarthritis, application of MSC-EV resulted in suppression of pain, reduction of inflammation, and improvement in subchondral bone architecture [108].

Additionally, in a mouse model of osteoarthritis, treatment with BMSC-derived EV containing miR-206 decreased the inflammatory cytokines TNF-α, IL-1β and IL-6. Besides, it increased the expression of genes encoding osteocalcin and bone-morphogenetic protein 2 (BMP2) in femoral tissues. EV produced an increase in alkaline phosphatase activity, mineralization, osteocalcin secretion, and proliferation, further inhibiting apoptosis, using osteoblasts from these mice in vitro. This effect was mediated by repression of the gene encoding E74-like factor 3 (*Elf3*) through miR-206 [109].

Another miRNA expressed in MSC-EV with proven anti-inflammatory capacity in osteoarthritis is miR-26a-5p. This miRNA interacts with the mRNA encoding prostaglandin-endoperoxide synthase 2 or cyclooxygenase-2 (PTGS2). Such gene expression was high, while that of miR-26a-5p was low, in osteoarthritis synovium samples and in synovial fibroblast cultures, treated with the inflammatory cytokine IL-1β. Increasing miR-26a-5p levels through joint injection of MSC-EV arrested disease progression through inhibition of inflammation, proliferation, and migration, while promoting cell apoptosis in an osteoarthritis rat model [110].

These data show that MSC-EV can act through different pathways to exert its anti-inflammatory effects in osteoarthritis. These include adenosine receptor activation of AKT, extracellular signal-regulated kinase (ERK), and adenosine monophosphate (AMP)-activated protein kinase (AMPK) phosphorylation [108]. Likewise, suppression of nuclear factor of kappa light polypeptide gene enhancer in B-cells inhibits, alpha (IκBα) phosphorylation, which prevents the translocation of nuclear factor kappa-light-chain-enhancer of activated B cells (NF-κB) into the nucleus and thus its transcriptional activity. The latter explains that treatment with BMSC-EV in chondrocytes from osteoarthritis patients, inhibited collagenase activity and downregulated genes encoding interleukins and COX2, which are targets of NF-κB. Interestingly, this effect on inflammatory parameters in chondrocytes occurred when conditioned medium from BMSC cultures, or their extracellular vesicles were used. On the contrary, the application of EV-free conditioned medium had no anti-inflammatory effects. Thus, the anti-inflammatory activity of BMSC was mainly mediated by EV secretion [111].

Osteoarthritis alters osteoblast metabolism, inducing them to produce inflammatory, catabolic factors and senescence [112]. These phenotypic changes can be reversed by adipose tissue derived MSC (ASC) EV [112]. The positive effect of MSC-EV on osteoarthritis can be enhanced by the application of low-intensity pulsed ultrasound (LIPUS). Thus, injection of 100 μL of a saline solution containing 40 μL of MSC-EV into the joint cavity of the knee, twice a week for four weeks, decreased inflammation and cartilage degradation. That was accomplished through inhibition of the NF-κB signaling pathway, activated by IL-1β in a rat model of osteoarthritis. This effect was significantly superior in animals that were additionally treated with LIPUS daily, relative to those treated with MSC-EV or LIPUS alone [113].

Additionally, preconditioning MSC with parathyroid hormone (PTH) 1-34 has been proposed to enhance the therapeutic effect of MSC-EV in osteoarthritis. Thus, BMSC cultures were treated with PTH for 6 h, and then, after changing the medium, maintained for 48 h before isolating EV from the conditioned medium. The results showed that MSC-EV derived from PTH pretreatment decreased the levels of IL-2, TNF-α and IL-6 inflammatory factors that also increased proliferation, migration, and extracellular matrix production, more intensively than MSC-EV without PTH pretreatment in an in vitro model of osteoarthritis in chondrocytes [92].

On the other hand, osteoporosis in diabetics has been associated with increased inflammation [114]. In this context, the anti-inflammatory capacity of MSC-EV could potentially be used to prevent or treat alterations of bone metabolism in people with diabetes. Indeed, intravenous injection of EV derived from adipocyte-derived mesenchymal stem cells in streptozotocin-induced diabetic and osteoporotic rats partially prevented a decrease in bone mineral density, as compared to untreated animals. That effect was associated with suppression of NLRP3 inflammasome activation in osteoclasts and subsequent inhibition of inflammatory cytokine secretion [115].

It should be noted that intense immune responses can lead to implant rejection in patients. To prevent this situation, several studies have evaluated the possible positive effect of MSC-EV. Thus, titanium implants covered with hydrogel containing MSC-EV enriched in miR-181b favored osseointegration, in a rat bone defect model. This effect was associated with the fact that these EVs promoted polarization in M2 macrophages. That decreased inflammation while facilitating osteogenesis through activation of the protein kinase C delta (PRKCD)/AKT signaling pathway [116]. Additionally, in vitro studies have shown that titanium nanotubes, together with EV derived from undifferentiated BMCS or induced to differentiate into osteoblasts for three days, have the ability to downregulate the expression of inflammatory cytokines in macrophage cultures. Likewise, they favored the migration and osteogenic differentiation of BMSC [117]. That supports the concept of using MSC-EV, together with biomaterial implantation, to promote bone regeneration.

### 5.2. Angiogenesis

When a bone injury or fracture is produced, vascularization plays an essential role in supplying minerals and growth factors as well as modulating bone formation and regeneration. Thus, blood vessels and vascular cells act as physical structures, around which bone formation starts. Additionally, they exert other essential functions in bone, such as creating niches for hematopoietic stem cells, or interacting with bone cells to regulate bone formation and growth [118]. Interestingly, communication between various cell types that form bone tissue, including osteoblasts, osteoclasts, endothelial and stem cells are closely linked to the promotion of osteogenesis and angiogenesis, which are mediated, in part, by EV secretion [119]. Endothelial cells improve the proliferative potential of MSC. These also produce endothelin-1 (ET-1), insulin-like growth factor (IGF), and bone morphogenetic protein-2 (BMP-2), promoting osteoblastic differentiation and therefore bone repair [120]. Previous studies have shown the potential of endothelial progenitor cell (EPC)-EV for human umbilical-cord vein endothelial cells (HUVEC) proliferation, migration and angiogenesis, through expression of exosomal miR-126 [121]. Likewise, numerous studies have shown that osteoblasts and their precursors can regulate vessel formation, through molecules contained in EV. This opens the door to the use of these EVs for clinical purposes in bone regeneration.

VEGF belongs to the group of factors that activate MSC-EV in bone, inducing angiogenesis and favoring bone regeneration. Indeed, it is one of the most important for the regulation of vascular development and angiogenesis. Moreover, it also acts on osteoblastic cells in bone, regulating their activity during bone formation and repair [122]. VEGF is critical for MSC-EV, promoting angiogenesis and bone formation. Thus, it has been found that application of anti-VEGF antibodies, together with BMSC-derived EV, inhibited the positive effects of EV on bone regeneration in a rat model of calvaria bone defect [123].

Among the angiogenesis-inducing molecules in MSC-EV are different classes of RNA, such as long noncoding RNA-H19 (lncRNA-H19). It is found in BMSC-derived EV and has been shown to regulate miR106a, functioning as a “sponge”, competing with mRNA for miRNA binding. Because miR106a targets the angiopoietin 1 (*ANGPT1*) gene, which is involved in activation of angiogenesis and osteoblastogenesis [124], intravenous injection of EV-BMSC carrying lncRNA-H19 increased ANGPT1 secretion, enhanced angiogenesis, and reversed bone mass loss in an osteoporotic mouse model [125].

Other RNA molecules included in MSC-EV with effects on angiogenesis are miRNAs. Among them are miR-23a-3p and miR-29b-3p. Both of them target the phosphatase and tensin (PTEN) homolog. Inhibition of PTEN by miR-23a-3p activates the AKT signaling pathway, which promotes angiogenesis and osteogenesis [126,127]. This has been demonstrated in vitro, as well as in an animal model of skull bone defect, where a bioglass scaffold with GelMA/nanoclay hydrogel containing UCMSC-derived EV enriched in miR-23a-3p was successfully applied [126].

On the other hand, hypoxia is one of the main signals inducing angiogenesis. Tissue damage may involve loss of vascularization. Generated hypoxia induces a response in cells to adapt to low O_2_ levels. That activates the formation of blood vessels to reverse the hypoxic situation. This response is mediated by the hypoxia-inducible factor (HIF) pathway. It is a transcription factor consisting of two subunits: HIFA and HIFB. Expression of the genes encoding HIFA is induced by hypoxia, although, interestingly, HIFB is constitutively expressed [128].

Under normoxic conditions, HIFA in the cytoplasm is hydroxylated and further degraded by the proteasome. While in hypoxia, hydroxylation is inhibited, and HIFA accumulates and translocates into the nucleus, where it forms heterodimers with HIFB. That way, it regulates the transcription of hundreds of genes. They are involved in numerous physiological processes, aimed at adaptation to hypoxia and tissue regeneration. Among these processes, angiogenesis, inflammation, glucose metabolism, migration, proliferation, apoptosis, and differentiation stand out [67,129]. In MSC, hypoxia induces the secretion of EV carrying molecules and factors involved in these physiological processes. For this reason, it has been proposed that MSC preconditioning under hypoxia, or HIF activation, can be used to increase the regenerative capacity of MSC-EV [67]. In this sense, at the level of bone regeneration, different studies have shown that MSC-derived EV with activated HIF has a greater therapeutic potential for the treatment of bone defects.

Thus, EV derived from hUCMSC maintained in hypoxia (1% O_2_) for 48 h had a greater angiogenic capacity in vitro and in vivo. Indeed, they accelerated bone fracture healing in a mouse model in relation to EV derived from cultures in normoxia [93]. Such authors showed that EV derived from hypoxia cultures was enriched in miR-126. Interestingly, that is an important inducer of angiogenesis, migration, and proliferation of endothelial cells, through repression of the sprouty-related enabled/vasodilator-stimulated phosphoprotein (VASP) homology 1 (EVH1) domain containing 1 (*SPRED1*) gene. Its encoded protein has anti-angiogenic activity, inhibiting the rat sarcoma (Ras)/Erk pathway, which is critical for activation of angiogenesis [93]. Another miRNA enriched in MSC-EV exposed to hypoxia is miR-210-3p. It promotes angiogenesis, facilitating bone regeneration through downregulating the gene encoding Ephrin A3 (*EFNA3*), which promotes phosphorylation and activation of the angiogenic PI3K/AKT pathway [130]. The angiogenesis-stimulating effect of BMSC-derived EV preconditioned in hypoxia can also be used as a possible therapeutic tool in the treatment of osteonecrosis of the femoral head, as recently shown in a rat model [95].

Another strategy to increase HIF synthesis has been to bioengineer the hypoxia-inducible factor 1-alpha (*HIF1A*) gene, to synthesize a functionally active protein that is not hydroxylated (and therefore is not degraded), under normoxic conditions. In this way, constitutive activation of the HIF pathway is achieved [131]. MSC have been transformed with such engineered gene, and their EV have been used for the treatment of femoral head necrosis in a rabbit model. Compared with EV derived from MSC carrying wild type HIF, injection into the necrosis zone of those from MSC, transformed with the mutant sequence increased angiogenesis and bone regeneration [131].

Similar results have been obtained with EV from BMSC preconditioned with dimethyloxalylglycine (DMOG), an inhibitor of prolyl hydroxylase responsible for HIF1A downregulation in normoxia. DMOG-treated BMSC synthesized higher levels of HIF1A [96]. Additionally, EV derived from these cells, applied on porous hydroxyapatite scaffolds on a critical-sized calvarial, favored bone regeneration. That was accomplished through the induction of angiogenesis to a greater extent in relation to EV from BMSC not preconditioned with DMOG [97]. These studies showed that overexpression of HIF produced EV with enhanced regenerative capacity.

However, for induction of the HIF pathway in MSC-EV target cells, it is not always necessary for MSC to overexpress *HIF*. Recently, it was observed that EV derived from human umbilical cord MSC increased both *HIF1A* and *VEGF* gene expression, in addition to tubular structure formation, proliferation, and migration, in HUVEC in vitro [132]. Those EV, applied inside a hydrogel on a fracture, accelerated bone regeneration in a rat model, mainly through the induction of angiogenesis [132].

Another factor present in MSC-EV positively influences angiogenesis, is the extracellular matrix protein nidogen 1 (NID1). It binds to collagen IV and laminin in the extracellular matrix. That generates the right conditions for endothelium establishment and the integrity of the vascular system [133]. BMSC-derived EV containing NID1 applied in a hydrogel in a rat model of femoral defects, increased angiogenesis and bone regeneration. Interestingly, this shows the importance of coupling angiogenesis and osteogenesis in bone formation [134]. In this regard, another advantage of using EV in bone regeneration is that, because they contain different types of molecules, they can act on different cell types and physiological processes at the same time. Besides, such molecules may work synergistically, multiplying instead of just adding their individual effects.

### 5.3. Differentiation

Modulation of differentiation of the main cells involved in bone metabolism (osteoblasts and osteoclasts) plays a fundamental role in both bone remodeling and regenerative processes [135,136]. Among the factors that regulate bone remodeling are EV secreted by different cell types in the bone [137]. Particularly relevant among them are those derived from BMSC for their physiological roles and potential clinical applications.

BMSC differentiation may be altered towards adipocytes instead of osteoblasts in various bone pathologies, such as osteonecrosis of the femoral head and osteoporosis [138]. Thus, it has been observed that BMSC from steroid-induced femoral head necrosis (SFHN) in rats differentiate more likely into adipocytes than osteoblasts, which is the opposite of what happens in BMSC from healthy rats. Interestingly, EV derived from the latter, applied to SFHN-BMSC cultures, activated osteoblast differentiation and inhibited adipogenesis. That highlights the clinical potential of these EVs for the treatment of such bone pathology [139].

It has also been observed that weekly intravenous injections of UCMSC-EV for two months maintained bone strength, preventing bone mass loss, in a mouse model of osteoporosis. That was accomplished through reduction of marrow adiposity and bone resorption, which favored bone formation [140]. This study identified that the anti-osteoporotic effect of UCMSC-EV was mediated, in part, by the C-type lectin domain containing 11A (CLEC11A) protein, also called stem-cell growth factor. Indeed, CLEC11A is an important osteogenic factor [141]. In vitro studies have shown that it has the ability to inhibit adipogenesis and promote BMSC osteogenesis, in addition to acting on the inhibitory effect of UCMSC-EV on osteoclast formation [140]. Different mechanisms have been proposed to explain how MSC-EV favors osteoblastogenesis. One of them is the activation of the PI3K/AKT signaling pathway. The rationale is that the inducing effect of UCMSC-EV on the differentiation of periodontal-ligament stem cells (PDLSC) into osteoblasts is inhibited by an inhibitor for this pathway (LY294002) [142].

BMSC-derived EV can also favor osteoblastic differentiation through the release of some miRNAs. This is the case with miR-335, which has been identified in mouse BMSC-EV [143]. Thus, injection of these EVs, on the first and eighth days after fracture, promoted bone regeneration in a mouse fracture model through activation of osteoblastic differentiation. This effect was mediated by the release of miR-335. It activated the osteogenic Wnt/β-catenin pathway by inhibiting vesicle-associated membrane proteins or synaptobrevin (VAMP)-associated protein B (VAPB) and C (VAPC); VAPB/C [143]. Another miRNA involved in osteoblast differentiation is miR-27. This is also found in BMSC-EV, and its application produced increased bone density, improved bone structure, decreased bone resorption markers, and decreased osteoclasts in a model of ovariectomized (OVX) mice with osteoporosis. This effect was, in part, due to the downregulating effect of miR-27 on the Dickkopf-related protein 2 (*DKK2*) gene, which encodes an inhibitor of Wnt/β-catenin [144].

Additionally, miR-1260a was also found to be involved in osteogenic induction. This miRNA was abundant in BMSC-EV, preconditioned with Fe_3_O_4_ nanoparticles (NP), in combination with a static magnetic field, to activate their osteogenic activity. Such miRNAs promoted osteogenesis through the repression of the histone deacetylase 7 (*HDAC7*) gene. That inhibited the activity of runt-related transcription factor 2 (RUNX2), which is a transcription factor critical for osteoblast differentiation [145]. Interestingly, miR-1260a also had the ability to increase angiogenesis in endothelial cells through repression of the gene encoding the anti-angiogenic factor COL4A2 [145]. Thus, miR-1260a-enriched EV has a high therapeutic potential in bone regeneration by jointly promoting osteogenesis and angiogenesis.

On the other hand, miR-196a is also abundant in human BMSC-EV. When these EVs were applied to human osteoblast cultures, they favored mineralization and expression of osteoblastic genes such as *RUNX-2*, alkaline phosphatase (*ALP*), osteocalcin (*OCN*), and osteopontin (*OPN*). Moreover, they induced bone formation in a rat model of calvarial defects [146]. Another notable miRNA in BMSC-EV is miR-22-3p and miR-935. Both stimulate osteogenic differentiation and bone formation in ovariectomized animal models. The former does so through suppression of the myelocytomatosis (MYC)/PI3K/AKT pathway by targeting alpha-ketoglutarate-dependent dioxygenase (FTO; name derived from “FaTsO”, due to its large size, deleted by the mouse “Fused Toes” (FT) mutation) [147]. The latter exerts its osteogenic effect through the repression of signal transducer and activator of transcription 1 (*STAT1*) expression [148].

Other RNA molecules contained in MSC-EV may also have osteogenesis-inducing capacities. Among them are lncRNA. For instance, the metastasis-associated lung adenocarcinoma transcript 1 (MALAT1) lncRNA. It can bind to miR-34c, preventing it from hybridizing with its target genes. They include the special AT-rich sequence-binding protein 2 (SATB2) [149]. It encodes a nuclear matrix protein that activates both transcription factor RUNX2 and activating transcription-factor 4 (ATF4), which are critical for osteogenic differentiation [150]. It has been demonstrated that human BMSC-derived EV containing MALAT1 lncRNA induced osteoblastic differentiation through such lncRNA, in vitro. Furthermore, when such BMSC-EV were injected into an ovariectomized mouse model of osteoporosis, they reduced bone mass loss by releasing MALAT1, which acted as a sponge on miR-34c. That allowed SATB2 activation and, consequently, osteogenesis [151].

MSC-EV can be enriched in miRNA with osteogenic activity by transfection. Qiu et al., 2021, transfected rat BMSC-EV with miR-150-3p, which is described to increase osteoblastic markers such as osteocalcin, alkaline phosphatase, and type I collagen during osteogenic differentiation [152]. Intravenous injection of miR-15-3p-enriched BMSC-EV in an osteoporosis model of ovariectomized rats decreased bone mass loss to a greater extent than when using non-transfected BMSC-EV. That was carried out through the promotion of proliferation, differentiation, and inhibition of apoptosis in osteoblasts [153].

Interestingly, preconditioning MSC under different culture conditions can enhance the osteoblastogenesis-inducing capacity of MSC-EV. Thus, EV derived from stem cells of adipose tissue were preconditioned with TNF-α inflammatory cytokine (1 ng/mL) for three days. Subsequently, they were maintained for another three days in the absence of such a cytokine. That way, they exhibited higher levels of WNT-3A protein than non-preconditioned cells [91]. Such ASC-EV from cultures preconditioned with TNF-α, promoted osteogenic proliferation and differentiation in human osteoblasts more intensely than non-preconditioned ones [91].

On the other hand, precoding of MSC with TUDCA, in addition to promoting an enrichment of anti-inflammatory factors in EV, as described above, also favored the presence of osteogenic factors such as ALP, RUNX2, BMP2 and bone morphogenetic protein-receptors (BMPR), together with miRNA, such as miR-136-5. That is involved in osteoblast differentiation through induction of the Wnt/β-catenin pathway [88]. On the other hand, in those EVs, miR-206, known for its ability to inhibit osteoblast differentiation [154], significantly decreased with respect to MSC-EV not treated with TUDCA [88]. Consequently, priming MSC with TUDCA induced the secretion of EV with a higher potential for bone tissue regeneration because of their enrichment in anti-inflammatory and osteogenic factors.

There are other strategies to enhance the secretion of MSC-EV with higher osteogenic capacity. For instance, obtaining EV from MSC maintained in osteogenic medium for 48 h [100]. These EV were shown to have a greater capacity to induce differentiation in osteoblast cultures than EV derived from undifferentiated MSC. Nevertheless, their effect on proliferation was lower [100]. However, both injections of differentiation-induced MSC-EV and undifferentiated MSC-EV showed positive effects on the reversal of bone loss in an ovariectomized mouse model. Thus, both types of EV had similar clinical potential for the treatment of bone pathologies, such as osteoporosis [100].

MSC-EV can also affect osteoclast differentiation. Thus, it has been observed that EV from ASC is enriched in OPG [155]. The latter is an inhibitor of RANKL and therefore, of osteoclast differentiation and activation [156]. These EV also carried miR-21-5p, which additionally promoted repression of osteoclast differentiation. That was accomplished through activin A receptor type 2A (ACVR2A) down-regulation [155]. Indeed, in a model of bone loss in OVX mice, systemic injection of ASC-EV increased bone formation through stimulation of BMSC migration and differentiation as well as inhibition of osteoclast differentiation. Therefore, ASC-EV has a high therapeutic potential for the treatment of bone pathologies, such as osteoporosis, due to its effect on reducing bone resorption [155].

Additionally, it has been recently described that MSC-EV-promoted inhibition of osteoclastogenesis can be enhanced through preconditioning of MSC in hypoxia. Thus, EV derived from dental-pulp stem cells (DPSC), maintained in hypoxia (1% O_2_) for 48 h, had greater capacity than EV produced in normoxia to inhibit osteoclast formation and promote M2 macrophage polarization. That was found in a rat model of lipopolysaccharide (LPS)-induced calvarial osteolysis. Such effect was mediated by miR-210-3p, which was enriched in DPSC-EV in hypoxia. It can repress expression of the gene encoding NF-κB1/p105, a key functional subunit of NF-κB. It also inhibits osteoclast differentiation and favors the appearance of the anti-inflammatory M2 phenotype in macrophages. These results suggest that DPSC-EV obtained under hypoxia have an important clinical potential, for treatment of pathologies associated with inflammatory osteolysis, such as osteomyelitis, periodontitis, peri-implantitis, septic arthritis and rheumatoid arthritis [94].

Another strategy to increase the osteoclastogenesis inhibitory capacity of MSC-EV is preconditioning such cells with bioactive-glass nanoparticles (BGN). Thus, BMSC-EV, maintained for 48 h in culture medium supplemented with BGN, had a greater inhibitory effect on osteoclastogenesis than BMSC-EV without BGN. That was observed both in vitro and in vivo, including an ovariectomized mouse model. This effect was mediated by an enrichment of non-coding repressor of nuclear factor of activated T cells (NFAT; non-coding repressor of nuclear factor of activated T cells (NFAT); NRON) lncRNA in BGN-treated BMSC-EV. NRON in osteoclast precursor may impede nuclear transfer of nuclear factor of activated T Cells 1 (NFATC1), and consequently inhibit their differentiation [99].

### 5.4. Migration

MSC migration is fundamental in the process of bone regeneration. Thus, in the first 24 h after suffering a fracture, there are two critical steps: (i) increase in proinflammatory cytokines, which intervene attracting MSC; and (ii) stimulation of angiogenesis at the damaged site, being fundamental for continuation and acceleration of bone regeneration [157]. Therefore, stimuli or treatments, promoting cell migration of osteoprogenitor cells to damaged sites, will favor bone formation, with potential clinical use. Interestingly, among the properties of MSC-EV are their ability to induce cell migration. Culture conditions can determine the effect of EV on cell migration. In the case of human BMSC, it has been described that EV derived from cultures induced to differentiate into osteoblasts for three days have a higher efficiency recruiting MSC than controls. Besides, MSC may decrease inflammatory responses at the site of bone defect, due to their immunomodulatory properties, therefore facilitating bone formation [86].

GMSC-derived EV also promoted migration of mouse calvaria MC3T3-E1 preosteoblasts, suggesting their use for periodontal treatments [158]. Additionally, periodontal ligament stem cell (PDLSC)-derived EV implanted at the site of bone damage using Matrigel, accelerated bone regeneration in vivo using a rat bilateral calvarial defect model. That was accomplished by stimulating cell infiltration, compared with treatment with Matrigel alone. This shows the ability of these EVs to induce osteoprogenitor-cell migration, which in this case was through activation of phosphorylation of AKT and extracellular signal-regulated kinase 1/2 (ERK1/2) [159].

On the other hand, the periosteum plays a pivotal role in initial callus formation and bone regeneration after trauma. That is in part due to paracrine processes, which promote migration and differentiation of preosteoblastic cells [160]. Considering the role of the periosteum in bone formation, stem cells from this tissue and EV from these cells have been isolated. The latter have a potent positive effect on BMSC proliferation, migration, and osteogenic differentiation in vitro. Furthermore, they have the ability to accelerate bone formation in vivo, as shown in a rat model of femoral defects. That was accomplished by stimulating the migration of osteoprogenitors to the fracture site [161]. Some interesting molecules were identified in these EVs. They included proteins such as collagen Type I, fibronectin, decorin, and biglycan. Likewise, miRNAs such as miR-191 are involved in cell proliferation, migration, and differentiation. That explain the osteoinductive effects of these EVs on bone regeneration [161].

Another source of EV with the capacity to affect osteoblast precursor migration, is perivascular stem cells (PSC). Interestingly, low concentrations of PSC-EV potently induce osteoprogenitor migration. On the other hand, high doses favored proliferation and differentiation. Such differential dose-dependent effects may be relevant in the presence of damage in vivo. Thus, curiously, cells at the site of injury would secrete EV, creating a gradient. Thus, in a more distant region from the damaged tissue, EV would be at a lower concentration, causing the migration of precursor cells towards the damaged region. In a nearby region, the concentration of EV will be higher, inducing the proliferation and differentiation of osteoprogenitors, regenerating damaged tissue [162]. Therefore, PSC-EV has an interesting potential in endogenous regeneration and regenerative medicine of bone. All these findings highlight the relevance of scientific knowledge of these phenomena, with clear clinical applications in translational medicine.

## 6. Incorporation of Mesenchymal Stem Cell-Derived Extracellular Vesicles into Biomaterials to Accelerate Bone Regeneration

Bone tissue regeneration is important for patient rehabilitation, especially in critical fractures, but remains a challenge in biomedicine. Fortunately, advances in knowledge of regenerative biomaterials open a new topic in the field of regenerative medicine. This way, the development of biomaterials has facilitated an interesting approach for the therapeutic purposes of bone-tissue regeneration. Indeed, they allow them to closely mimic key aspects of bone tissue physiology. That can be accomplished thanks to their beneficial properties. However, it is not only important that these biomaterials are effective and safe. They must also be cost-effective and convenient to be clinically and commercially successful [129].

Implantation of biomaterials to promote bone formation has several requirements. Thus, in order to be effective, they must promote migration, proliferation, and differentiation of different cell types responsible for the regenerative process within the implant. They must also allow the formation of vessels that supply oxygen and nutrients, which are necessary for the development of new tissues. That can be accomplished by incorporating MSC-EV into biomaterials. As described above, the former has the ability to modulate inflammation, induce angiogenesis, promote osteoprogenitor-cell migration, and favor osteogenic differentiation. Therefore, their presence in biomaterials can significantly accelerate bone regeneration and thus facilitate implant success. For this reason, numerous studies have developed different types of biomaterials with the capacity to encapsulate MSC-EV in order to accelerate bone regeneration.

Biomaterials used as vehicles to encapsulate MSC-EV include hydrogels. These are formed by a three-dimensional network of hydrophilic polymers. Such materials must have specific useful properties. They include porosity, wetting and gas exchange capacity, the formation of matrices that facilitate cell proliferation and migration, biocompatibility, and biodegradation, as well as the capacity to incorporate drugs, bioactive compounds, EVs and even cells. These properties have allowed them to have multiple applications in medicine, such as cell therapy, wound treatment, and drug delivery, among others [163]. Specifically, in the treatment of bone defects, the use of a hydrogel based on chitosan, a natural polysaccharide, with the addition of β-glycerophosphate to make it injectable and thermosensitive, has been studied in a rat calvarial defect model. BMSC-EV encapsulated in this hydrogel promotes bone healing, through increased angiogenesis. This effect was mediated by EV containing miR-21, which: (i) downregulated the prout-receptor tyrosine kinases (RTK) signaling antagonist 2 (*SPRY2*) gene; and (ii) upregulated genes encoding angiogenic factors such as VEGF, FGF, and ANGPT1 [98].

Recently, an alginate-based hydrogel has been developed in which magnetic cobalt ferrite and EV nanoparticles, derived from adipose tissue MSC, have been incorporated. In vitro studies with the resulting hydrogel showed that it increased AdMSC osteogenic proliferation and differentiation, to a greater extent than hydrogel alone or hydrogel with AdMSC-EV without magnetic nanoparticles [164]. This effect was stimulated by a static magnetic field (SMF). Indeed, magnetic nanoparticles have high clinical potential in regenerative medicine [165]. Even more, combining them with MSC-EV, which synergistically increases the regenerative capacity of such nanoparticles, is a promising therapeutic strategy for the treatment of bone pathologies [164].

However, the low consistency of hydrogels could represent a limitation of this type of biomaterial, for the treatment of more severe bone defects. Therefore, combination with scaffolds, which provide a higher consistency, is an interesting approach to overcome such limitations. Indeed, this possibility has recently been evaluated encapsulating UCMSC-derived EV in a hyaluronic-acid hydrogel, which was subsequently combined with nanohydroxyapatite/poly-ε-caprolactone (nHP) scaffolds. Application of the resulting biomaterial significantly increased bone regeneration in a rat calvarial defect model. At the histological level, the expression of angiogenic and osteogenic markers was higher than in untreated animals or when treated with biomaterials without EV [51].

In vitro studies showed that UCMSC-EV induced angiogenesis, migration, and proliferation of endothelial progenitor cells through miR-21. It inhibits the neurogenic locus notch homolog protein 1 (NOTCH1/delta-like 4 (DLL4) pathways, favoring expression of genes encoding vascular-endothelial growth factor A (VEGFA) and HIF1A angiogenic factors [51]. Therefore, application of EV combined with scaffolds and hydrogels generated a material with good mechanical properties for the repair of bone defects, good encapsulation capacity, and sustained release of EV. That compensates for the deficiencies of scaffolds or hydrogels alone.

Other scaffolds have been studied to release EV at sites of bone defects or fractures, with the aim of accelerating bone formation. Among them are those based on poly(lactic-co-glycolic acid) (PLGA). These biomaterials are characterized by being biodegradable and having appropriate mechanical strength. Therefore, they have been widely used in tissue engineering, including applications to bone regeneration [166]. Immobilization of MSC-EV on PLGA scaffolds has been achieved by polydopamine-coating PLGA, creating PLGA/polydopamine (PDA) scaffolds. Interestingly, EV derived from AdMSC cultures, after two days of osteogenic induction, had more osteoinduction capacity on BMSC in vitro than those obtained from undifferentiated cells or those induced to differentiate for a longer time. These EVs were incorporated into PLGA/PDA scaffolds. Application of the resulting biomaterial in a mouse critical-sized calvarial defects favored bone regeneration with respect to implantation of scaffolds without EV [167]. Therefore, this result shows the importance of incorporating MSC-EV in scaffolds to increase osteoinduction of these biomaterials for the treatment of important bone defects.

## 7. Conclusions and Perspectives

MSC-EV are considered part of the mechanism by which MSC have regenerative properties. Therefore, numerous studies have evaluated the clinical potential of MSC-EV in regenerative medicine [41]. Current research, mainly at the preclinical level, supports the development of therapeutic strategies that incorporate MSC-EV for the treatment of pathologies related to bone metabolism, regeneration, and formation. They include difficult-to-heal fractures, osteoporosis, osteonecrosis, and osteoarthrosis, among others (Table 2). Cell-free therapies, based on the use of MSC-EV, have advantages over other, more conventional treatments. That is due to the complex content of EV, which may include different factors and bioactive molecules. Besides, they can intervene throughout the entire process of bone regeneration. That spans from the early stages of inflammation to remodeling. Moreover, as mentioned above, the clinical use of EV has a number of advantages over cell therapy. They include ease of collection, handling, and storage, a lower potential for tumorigenicity, and greater versatility in the evaluation of dose and efficacy, which are similar to those of conventional pharmaceuticals.

On the other hand, their stability and the possibility of incorporating them in different types of biomaterials allow a better adaptation to the characteristics of the pathology to be treated. For example, for the treatment of bone fractures, they can be incorporated into biomaterials that are subsequently implanted in the fractured zone. For the treatment of osteoporosis, which has a systemic character, they can be applied by intravenous injection.

However, although preclinical studies published in trials show very encouraging results in animal models of different bone pathologies, their clinical translation is very scarce. Indeed, very few clinical trials are currently evaluating the use of MSC-EV in bone formation or regeneration in humans. Thus, searching the ClinicalTrials.gov (accessed on 28 October 2022) database for the terms extracellular vesicles and bone, or exosomes and bone, only identified four trials that aim to evaluate the effect of MSC-EV on different bone pathologies at the time of writing (Table 3). Of these trials, only one was in the process of recruiting patients; two had not started, and one had not been reported for two years.

Therefore, it is necessary to advance in the development of clinical trials in order to know the real clinical potential of MSC-EV in bone regeneration. It is required to evaluate their efficiency and possible side effects in patients with bone diseases in the coming years. Besides, in parallel, it is necessary to advance in the optimization of MSC-EV production and isolation techniques. Currently, they are very varied, according to the different studies published. In this regard, it is needed to identify/standardize the following aspects: (i) the source of MSC used; (ii) possible modifications through bioengineering, to make them more stable and proliferative in vitro; and (iii) the most appropriate culture or preconditioning conditions, depending on the pathology to be treated. It is also necessary to advance in production systems, using bioreactors that allow obtaining sufficient quantities of EV for clinical use. This must be accompanied by better knowledge of the recommended doses and by standardizing the degree of purity of EV for clinical purposes.

On the other hand, it is also necessary to continue advancing our knowledge of the mechanism by which MSC-EV exert their action on bone metabolism. Many molecules responsible for the regenerative properties of EV, including miRNA and growth factors, have been identified. Yet, there is much variability depending on the published study. Therefore, it would be useful to know which set of factors or molecules should be enriched in MSC-EV, allowing for optimal clinical effectiveness. Furthermore, this knowledge could be used in the future for the development of artificial EVs. That should allow for the generation of EV with defined compositions, including new drugs for the treatment of bone diseases. This is important because, as a consequence of the multiple factors found in the MSC-EV cargoes, their application could produce undesirable secondary and pleiotropic effects. It is necessary to take this into account, mainly for possible applications in pathologies such as osteoporosis, in which treatments are of long-term duration.

In summary, the use of MSC-EV in bone regeneration has significant therapeutic potential. However, for safety and routine clinical use, progress still needs to be made in key aspects related to their production and isolation. Likewise, in relation to their mechanisms of action, doses and treatment guidelines. In addition to better knowledge of possible side effects. For all that, it is necessary to increase the development of clinical trials, to properly evaluate their efficacy in different bone pathologies.

## Figures and Tables

**Table 1:** Effects of MSC preconditioning on EV cargos and responses during bone remodeling.

MSC Preconditioning	Conditions	Cargo	Response	References
TUDCA	2.5 µM	↑ Anti-inflammatory cytokines (IL1RN, IL6, IL10 and IL11) ↑ Anti-inflammatory miRNA (miR-145-5p) ↓ Inflammatory miRNA (miR-423-3p) ↑ Osteogenic factors (ALP, RUNX2, BMP2 and BMPR) ↓ Anti- osteogenic miRNA (miR-206) ↑ Osteogenic miRNA (miR-136-5)	Improve bone-tissue regeneration	[87,88]
Inflammatory cytokines TNF-α	72 h 20 ng	↑ Anti-inflammatory miRNA (miR-15b, miR-19b and miR-22)	M1 macrophage polarization into M2 Produce greater bone formation and reduce inflammation	[89]
	–	↑ CD73 and miR-1260b ↓ WNT5a expression and RANKL	M1 macrophage polarization into M2 Reduce inflammation and osteoclastogenesis	[90]
	72 h 1 ng/mL	↑ WNT-3A protein	Greater osteogenic proliferation and differentiation	[91]
PTH 1-34	6 h + 48 h before isolation	↓ Inflammatory factors (IL-2, TNF-α and IL-6)	Increase proliferation, migration and extracellular-matrix production	[92]
Hypoxia	1% O_2_ 48 h	↑ Angiogenic miRNA (miR-126 and miR-210-3p)	Greater angiogenic capacity Accelerate bone-fracture healing M1 macrophage polarization into M2 Inhibition of osteoclastogenesis	[93,94]
	2% O_2_ 48 h	↑ Angiogenic miRNA (miR-210-3p)	Angiogenesis stimulation Improve osteonecrosis of femoral head	[95]
DMOG	-	-	Greater angiogenic capacity Bone regeneration	[96,97]
Fe_3_O_4_ nanoparticles	Combination with static magnetic field	↑ Osteogenic miRNA (miR-1260a)	Promote osteogenesis and angiogenesis	[98]
BGN	48 h	↑ (NFAT; NROM) lncRNA	Inhibition of osteoclastogenesis	[99]
Osteoblast differentiation	Osteogenic medium 48 h	-	Enhance differentiation of osteoblast cultures Lower proliferation capacity	[100]
	Osteogenic medium 72 h	-	Higher recruitment of MSC Immunomodulatory properties Improve bone formation	[86]

**Table 2:** Potential of MSC-EV for treatment of bone diseases on preclinical animal models.

Disease	Model	MSC Culture	EV Isolation	Application	Ref.
Osteoporosis	Sprague Dawley rats	Rat ADMSC Culture media not specified 48 h	Ultracentrifugation	Mode: intravenous injection EV dose (µg/µL): 0.1, 0.2, 0.5 and 1.0	[115]
	C57BL/6J and 129P2-Cbstm1Unc/J (CBS KO) mice	Mouse BMMSC FBS-free medium 24 h	Ultracentrifugation	Mode: intravenous injection EV dose: 100 µg/mL	[125]
	C57BL/6 mice	Human UCMSC EV-free FBS medium 48 h	Ultracentrifugation	Mode: intravenous injection EV dose: 100 μg/100 μL in PBS	[140]
	C57BL/6 mice	Human BMMSC EV-free FBS medium 48 h	Ultracentrifugation	Mode: intravenous injection EV doses: 100 μL/mL	[144]
	BALB/c mice	Human BMMSC FBS-free medium 48 h	Ultracentrifugation	Mode: injection into the marrow cavity of femur EV dose: 25 μg/mL	[151]
	Sprague Dawley rats	Rat BMMSC transfected with miR-150-3p FBS-free medium 24 h	exoEasy Maxi Kit	Mode: intravenous injection EV dose: 100 and 200 μg	[152]
	ICR (CD-1) mice	ADMSC FBS-free medium 24 h	Multi-filtration system based on tangential flow filtration	Mode: intravenous injection EV dose: (1 × 10^8^ or 5 × 10^8^ particles/100 μL PBS)	[155]
Osteo-Arthritis	BALB/c mice	Human BMMSC and ADMSC FBS-free medium 48 h	Exocib Exosome Extraction Kit	Mode: intravenous injection EV dose: 100 μg/mL	[103]
	Sprague Dawley rats	Human ESC (E1-MYC 16.3) FBS-free medium 72 h	high-performance liquid chromatography (HPLC) fractionation	Mode: intra-articular injection EV dose: 5 μg/mL	[108]
	C57BL/6 mice	Mouse BMMSC—transfection of miR- 206 culture media not specified 72 h	Ultracentrifugation	Mode: intra-articular injection EV dose: not specified	[109]
	Wistar rats	Human BMMSC - EV-free FBS medium 48 h	ExoQuick-TC Re- agent Kit (EXOTC10A-1)	Mode: intra-articular injection EV dose: 400 μg/mL	[110]
	Sprague Dawley rats	Rat BMMSC EV-free FBS medium 48 h	Ultracentrifugation	Mode: intra-articular injection EV dose: 2 μg	[113]
Fracture	Mice	Human UCMSC Hypoxia (1% O_2_) Preconditioned EV-free FBS medium 48 h	Ultracentrifugation	Mode: intravenous injection EV dose: 200 μg/200 mL in PBS	[93]
	Wistar rats	Human UCMSC Culture media not specified	Ultracentrifugation	Mode: intravenous injection EV dose: 1 × 10^10^ particles	[132]
	C57BL/6 WT mice	Murine BMMSC EV-free medium 48 h	Ultracentrifugation	Mode: intravenous injection EV dose: 100 μg	[143]
	Sprague Dawley rats	Rat SMC and PCCM FBS-free medium 72 h	Ultracentrifugation	Mode: intravenous injection EV dose: 200 μg/100 μL in PBS	[161]
Critical-Sized Bone Defects	C57BL/6J mice	Human BMMSC TNFα preconditioned 72 h FBS-free medium 24 h	ExoQuick-TC Reagent (System Biosciences)	Mode: collagen scaffold EV dose: 4.5 × 10^9^ particles	[89]
	C57/BL6 mice	Human BMMSC Transfection of miR-181b FBS-free medium 72 h	Ultracentrifugation	Mode: intravenous injection EV dose: not specified	[116]
	Sprague Dawley rats	Human BMMSC -DMOG preconditioned Culture media not specified 48 h	Ultracentrifugation	Mode: hydroxyapatite scaffolds EV dose: 200 μg/100 μL in PBS	[97]
	Sprague Dawley rats	Human BMMSC Culture media not specified 24 h	Ultracentrifugation	Mode: hydrogel EV dose: 5 μg/mL	[146]
	Sprague Dawley rats	Human PDLSC FBS-free medium 24 h	Ultracentrifugation	Mode: Matrigel matrix EV dose: 60 µg/10 µL in PBS	[159]
	*Rattus* sp.	BMMSC- EV-free FBS medium 48 h	Ultracentrifugation	Mode: chitosan hydrogel EV dose: 1 × 10^8^ particles/mL	[98]
	Wistar rats	UMSC and HEK293 cells Culture media and time not specified	Ultracentrifugation	Mode: hyaluronic acid hydrogel, combined with nanohydroxyapatite/poly-ε-caprolactone (nHP) scaffolds EV dose: 1 × 10^12^ particles/mL	[51]
	BALB/C mice	Human ADMSC differentiated into osteoblast-EV-free FBS medium 0, 2, 4, 7 and 14 days	Ultracentrifugation	Mode: PLGA/PDA scaffolds EV dose: 25 µg/mL	[167]
Osteonecrosis	Rabbits	Rabbit BMMSC Transfected with HIF1aMU and Adv-HIF1aWT FBS-free medium 24 h	Total Exosomes Isolation Kit	Mode: femoral-head injection EV dose: 10 µg/mL	[131]
	Sprague Dawley rats	Rat BMMSC Hypoxia (2% O_2_) preconditioned EV-free FBS medium 48 h	Ultracentrifugation	Mode: intravenous injection EV dose: 50 µg/mL	[95]

**Table 3:** Clinical trials evaluating the effects of MSC-EV for the treatment of different bone pathologies.

ID Principal Investigator	Title	Status	Conditions	Interventions	Main Results
NCT05520125 PI: Dr. Andrei Hancharou	Treatment of Patients with Bone Tissue Defects Using Mesenchymal Stem Cells Enriched by Extracellular Vesicles	Not yet recruiting	Segmental Fracture—Bone Loss	Biological: Mesenchymal stem cells enriched by extracellular vesicles Other: Standard treatment of bone defects	Primary Outcome: adverse effects associated with the therapy and Percent of completely recovered patients with segmental bone tissue defects
NCT04998058 PI: Dr. Eduardo R Teixeira	Autogenous Mesenchymal Stem Cell Culture-Derived Signalling Molecules as Enhancers of Bone Formation in Bone Grafting	Not yet recruiting	Bone Loss, Osteoclastic Bone Loss, Alveolar Alveolar Bone Loss Alveolar Bone Atrophy Grafting Bone	Procedure: Maxillary sinus floor elevation grafting with synthetic bone substitute.	Primary Outcome: assessment of changes in bone density and quantity.
NCT04270006 PI: Dr. Ebtehal Mohammed	Evaluation of Adipose Derived Stem Cells Exo.in Treatment of Periodontitis	Unknow status	Periodontitis	Biological: adipose derived stem cells exosomes	Primary Outcome: change in gingival inflammation, pocket depth and attachment and bone level.
NCT04223622 PI: Chiara Giannasi	Effects of ASC Secretome on Human Osteochondral Explants	Recruiting	Osteoarthritis	Biological: ASC secretome	Primary Outcome: validation of the cell-free approach based on the use of ASC secretome on an ex vivo OA model by evaluation of cell viability, histological features and gene/protein expression of cartilage and bone biomarkers

## Data Availability

Not applicable.
